# Analectic

**Published:** 1828-07

**Authors:** 


### MISCELLANEOUS INTELLIGENCE.

---

### I. ANALECTIC.

1. Biancini *on the communication between the blood vessels of the Uterus and Placenta.*—M. Biancini injected the blood vessels of a woman who died in child-bed with inertia of the womb, the placenta being still undetached from the uterus. He found the injection in the vessels of the chorion and amnios; and having examined the tortuous arteries of the uterus, he found that they penetrated the tissue of the placenta, spread themselves over its membranes, and deposited the injection in the cells described by Hunter and Meckel. In a young woman who died eight days after delivery, a small portion of placenta retained its adherence to the womb. In this case, injection forced into the uterine arteries, not only passed into the residuary portion of placenta, but also was extravasated into the cavity of the womb, and into the vagina, from the lacerated ends of what he calls utero-placental arteries.

But the most extraordinary facts in support of M. Biancini's opinions, are those elicited from the examination of the subject in brutes. Not only did he find in cats, that the colouring matter of fine injection passed to the umbilical vein, but he actually found the vermilion to have tinged the vena cava superior, and some of its smallest ramifications. Such at least is the statement in the Journal des Progres. We see not very readily why the vermilion should be found in the *upper cava.*

M. B., to make assurance doubly sure, next inverted the modus operandi. He injected mercury into the umbilical arteries of a calf, and found not only that it passed into the placenta, but directly into the uterine veins, by nine short cylindrical branches, which he calls placento-uterine veins.—*N. A. M. & S. Journ. for July,* 1828.

2. *New Views of the Fœtal Circulation.*—We find in the Bulletin des Sciences Medicales for February, a notice of the work of Dr. H. Fr. Kilian, published at Carlsruhe, in 1826.

In the work is contained an account of the development of the heart of the fœtus, from its first appearance until the third and fourth month of gestation. M. K. asserts that, in the early stages of pregnancy the inferior cava opens both into the right and left auricles, by distinct mouths. At the commencement, the *left* opening is much the largest; but towards the close of the ninth month, the *right* has become much the largest. The valve of Eustachius and the valve of the *foramen ovale* constitute part of the inferior cava, and are to be considered as duplicatures or prolongations of the internal coat of the vein. These valves appear to be formed at the same epoch; but the valve of Eustachius is more rapidly and perfectly developed than the other. The valve of Eustachius, which principally constitutes the orifice of the inferior cava of the right auricle, serves to direct the two great columns of blood brought by the superior and inferior venæ cavæ, so that one may pass before the other, without interference or mixture. The valve of the foramen ovale, or rather the left half of the lower cava, is a *vascular branch:* its use is gradually to effect the exclusion of the *cava inferior* from the left auricle, and favour its passage or transfer to the right auricle. The septum of the heart is not perforated; the foramen ovale passes behind it through a notch. There is, properly speaking, no pulmonary artery in the fœtus. The trunk that bears the name, is continuous with the *canalis arteriosus*, and constitutes the origin of a vessel which goes to the lower half of the body, and may be named *abdominal aorta*, to distinguish it from the cerebral aorta which rises from the left ventricle; so that the fœtus has a double arterial circulation, one derived from the right ventricle, which goes to the body, and the other from the left, which supplies the head.

M. Kilian's doctrine of the fœtal circulation is as follows. Blood comes from the cells of the placenta, through the umbilical vein to the liver; it is there divided into two unequal portions, the largest of which circulates in the vessels of the liver, while the smaller portion goes through the *ductus venosus* into the vena cava. Here the blood of the ductus venosus, of the liver, and of the lower extremities, is mixed. Just before it enters the heart, the current is divided, as above stated; one stream enters the right at the same moment that the other enters the left auricle. Upon the contraction of the auricles, the ventricles are filled, and, in their turn, they throw it out *without any mixture taking place;* for that of the right goes to the descending aorta exclusively, and that of the left is distributed upwards. The author asserts, that if different coloured injections be thrown into the aorta and pulmonary arteries of a fœtus, at the same moment, no mixture of colours will take place. The rest of the circulation, namely back into the veins, he explains in the ordinary manner.—*Ibid.*

3. *Puerperal Peritonitis cured by Ice.*—A female was seized with severe symptoms of peritonitis on the fourth day after parturition. She was bled to ℥16; ice water for drink, and ice to the abdomen. In three hours, the severity of symptoms was diminished; thirst and vomiting had disappeared; abdomen less tumid, and pressure less painful. In the evening, amelioration still greater; the belly quite soft; a natural stool occurred, and the patient had some sleep. The application of ice was alone continued, and next day perspiration came on. The mammæ filled with milk; the lochiæ soon appeared, and in four days no symptom of peritonitis remained.—*Ibid.*

4. *Congenital absence of Sensation and voluntary Motion in a Female.*—This girl was born rather prematurely. She gave no sign of the usual instinctive desire for the breast; was, with difficulty, taught to suck, and appeared to be very feeble. One eye was smaller than the other. A few weeks after birth, she was seized with convulsions: no medical prescriptions had any power to restrain them, and they exceeded a hundred in twenty four hours. As she continued to grow in length, the defects in motion and sensation became more apparent. Blindness came on; she became quite deaf, and never had a second dentition. Taste seemed tolerably perfect. She never acquired any thing like the power of voluntary motion, and attained the 17th year without having held up her head, raised her hand to her mouth, or set her foot to the ground. She never uttered any thing like an articulate sound, and a feeble cry or whine was believed to indicate the want of food. When fed with sweet articles, the countenance exhibited an expression of satisfaction.

She never had a stool without medicine, and never acquired any command over [illegible] sphincters. On the day when she completed her 17th year, s[illegible]d in a manner so gradual, that the attendants thought her asleep, when she was really dead.

With regard to the morbid appearances on dissection, the following alone seem to possess any interest.

On removing the brain from the basis of the scull, so as to expose the nerves, he could trace each pair taking their usual course to their destination; and they all appeared firm and healthy, as in the most intelligent individual. But in the base of the scull itself not a vestige of the dura mater was to be seen. Its place was supplied by a thin semitransparent membrane, very lax and irregular, so that it afforded no protection to the nerves in their exit from the scull.

On the back part, likewise, the whole or the greater part of the *tentorium* was deficient, thus allowing the whole weight of the brain to rest on the cerebellum.

The author's opinion is, that the girl's deficiencies were occasioned by the want of dura mater on the base of the scull, the mass of brain being thereby allowed to press on the nerves of sense as they passed through their separate foramina, and thus their vital principle became destroyed; and that the cerebellum, not protected by a tentorium, was also pressed upon by the cerebrum, which also suffered in its turn.—*Ibid.*

5. *Tetanus.*—M. Lepelletier, surgeon in chief to the hospital at Mans, has always found, when dissecting cases of traumatic tetanus, that there were very visible traces of phlegmasia in the meninges of the medulla spinalis, especially of the arachnoid at the roots of the nerves, and over the latter, during their progress through the spinal canal. After a case of well characterized tetanus with trismus, opisthotonos, &c., the sacral nerves, especially the sciatic, exhibited spots and numerous striæ of a vermilion red. When divided longitudinally, the nerves presented the same striæ; and when cut transversely, exhibited a multitude of bright red specks, which were the orifices of vessels. All these appearances existed in the neurilema, either in the portion covering the nerve, or in that which separated its different fibrillæ; but principally in the former. If one of these divided nerves was pressed, it gave issue to a considerable quantity of blood. The medulla spinalis, when simply laid bare, exhibited bluish striæ, and its tunics seemed distended by a fluid. The fibro-cellular membrane, (?) when incised throughout its whole length, contained, on the inside, numerous red patches, especially at the origin of the nerves: and the arachnoid was particularly remarkable for a vascular network very strongly marked.

The arachnoid of the brain was considerably injected, but presented neither patches nor adhesions.

In another case of tetanus, the disease was provoked by the left arm having been crushed; and the first tetanic symptoms were manifested in the arm. The neurilemas, principally of the ulnar and median nerves, were red, and inflamed quite up into the axillary plexus. The spinal pia mater and arachnoid showed mar[illegible]ngly marked inflammatory spots; but all these appearances [illegible]onfined to the side which had received the injury. The strongest characteristic marks of inflammation were found in those parts of the medulla spinalis which were most in connexion with the brachial nerves, the first point of departure of the neurilematic inflammation. It is exactly in those muscles, whose nerves arise from the portion of the spinal marrow already pointed out, that the propagation of the disease was observed. Thus the respiratory muscles exhibited the tetanic stiffness in all its force; and the patient was several times on the point of suffocation from the mechanical suspension of respiration; while the trismus was only consecutive, and the jaws could be separated to the very moment of death.

The resemblance of these views to those of Dr. Saunders, of Edinburgh, will be noticed.

M. Lepelletier concludes, from these and other cases, that tetanus is an inflammation of the neurilema of the nerves and spinal marrow; (would he not include the trigeminus of the head in the case of trismus?) that traumatic tetanus spreads from the cirumference to the centre, thus attacking the nervous tree from the branches to the trunk; that tetanic patients die from exhaustion, with the exception of those instances in which respiration is actually suspended long enough to produce a true asphyxia; and, finally, that the rational treatment for this formidable malady must consist in lowering the vitality of the injured organ in particular, and of the whole economy in general.—*Ibid.*

6. *Mercury absorbed into the System.*—The analysis lately made in the chemical laboratory of the faculty of Paris, of the mammary and salivary glands, mesentery and large intestines, of a young woman who had fallen a victim to a puerperal peritonitis, and who during the disease had been subjected to large mercurial frictions (12 oz.

5 dr. in the course of twelve days), exhibited mercury in its metalic state in these organs. This result, though opposed to the assertions of many chemists and a great number of physicians, confirms the researches of Fourcroy, Dumeril, Orfila, and Cruvelhier, who have discovered mercurial globules in the bones, cerebral substance, and nerves; and no longer allows us to doubt of the fact, notwithstanding the warmth with which it has been, at different epochs contested, and the ridicule attempted to be thrown on it.—*Ibid.*

7. *Amputation of the Lower Jaw.*—This operation has now been so frequently executed with success, that surgeons are directing their attention to the best means of preventing, as much as possible, the subsequent deformity. The enterprising and active surgeon, M. Lisfranc, in November last, extirpated the left portion of the lower jaw from the chin to the ramus, for a sarcomatous tumour involving the bone, extending under the tongue, and contaminating, in a slight degree, the lymphatic glands. To prevent the deformity, M. Lisfranc, instead of making the ordinary transverse incision across the cheek, mad[illegible] perpendicular cut from the middle of the free border of the lip to the base of the jaw, dividing all the soft parts to the bone. The incision was continued an inch further in the same direction; it was then made transversely from before, backwards to a point one quarter of an inch below and before the angle of the jaw, and extending only through the skin and cellular tissue. The flap thus formed was dissected upwards, and the bone and diseased mass extirpated in the usual manner, without any troublesome hemorrhage. The fluids from the mouth and wound had a free outlet in a depending position; part of the wound healed by the first intention, part by granulation; and a fistula, kept up by the discharge of saliva, &c., was formed on the side of the neck. M. Lisfranc succeeded in curing it, by placing in the mouth, at a point corresponding to the external orifice, a small piece of sponge which absorbed the fluids that retarded the cure. The sinus then healed in four days. Little deformity resulted; the cicatrix being nearly covered by a cravat. The left side of the jaw is but slightly depressed; while the right side has deviated a little to the left, but can easily be replaced in its ordinary position. The left ramus of the bone is carried a little inwards. The patient speaks with much distinctness and can take solid food.—*Ibid.*

8. *Writ for the Medical Convention of* 1830.—Whereas the Convention that was held at the City of Washington, in the month of January, 1820, for forming a Pharmacopoeia for the United States of America did resolve that the President of that Convention should, on the 1st day of January, 1828, issue Writs of Election to the several incorporated State Medical Societies, in the northern, middle, southern, and western districts of the nation, requiring them to ballot for three Delegates to a General Convention, to be held at Washington, on the 1st day of January, 1830, for the purpose of revising the American Pharmacopoeia; and whereas the several Institutions, as aforesaid, are, by the same authority, requested to forward to the President, on or before the first day of April, 1829, the names of the three persons so chosen, with sundry other provisions contained in the historical introduction to the work, to which the reader is referred:

Now, therefore, I SAMUEL L. MITCHILL, by virtue of the power vested in me by the Convention of 1820, do hereby give notice to all the incorporated Medical Societies, Colleges of Physicians and Surgeons, Medical Schools, and Faculties of Universities and Colleges, and all other authorized Bodies, that they choose proper persons to represent them in the General Convention to be held in January, 1830, for revising the Pharmacopoeia.

Given under my hand, this first day of January, 1828, at the City of New York.

SAMUEL L. MITCHILL, *President.*

9. *Treatment of Poisoned Wounds.*—M. ANTOINE VERNIERE has very recently announced to the Academy of Sciences, a new method of treating poisoned wounds, which is, to say the least of it, ingenious, and philosophically deduced from the late discoveries in the physiology of absorption, and which, if it stands the test of future experiment, will render the treatment of external poisoning exceedingly simple. The reader who is acquainted with the progressive researches of M. Magendie on venous absorption, will remember that physiologist discovered that absorption is impeded, or even entirely prevented, by producing an artificial state of venous plethora, by injecting tepid water into the veins. M. Verniere's method of cure consists in taking advantage of this discovery, establishing a general venous plethora, and then removing the plethora as nearly as possible from that part only where the poison had been introduced, by opening a vein proceeding from the part. Thus, three grains of the alcoholic extract of nux vomica were thrust into the paw of a dog, and a tight ligature was without delay tied round the limb. Warm water was then very slowly injected into the jugular vein, to as great an extent as the animal could safely bear, and the ligature was removed. Half an hour was then allowed to elapse, which, in ordinary circumstances, would have been much more than sufficient to enable the poison to act. The ligature was next replaced, but so as to obstruct the venous circulation only, just as in performing the operation of venesection; the principal vein of the limb was then opened immediately under the ligature, and the blood which flowed out was collected. The blood was then carefully washed away, a little more was allowed to issue, and the ligature was finally removed. None of the symptoms of poisoning with nux vomica ensued, and the slight signs of venous plethora which remained were removed by a bleeding from the jugular vein. The blood which had been withdrawn from the vein of the fore-leg, when cautiously injected into the vein of another dog, caused violent tetanus and almost instant death.

The explanation given by M. Verniere of the mode in which this operation effects the removal of the poison, is the following:—"The only blood which can become impregnated with the poison, is that which flows through the open vein; for it is only that vein and its branches which are relieved from the artificial plethora, and so rendered capable of carrying on the function of absorption. On the other hand, by means of the ligature, the current of the blood taking place from the artery only towards the opening in the vein, the poison introduced into the vessels is forced to follow the course of the blood, and is consequenrly thrown out of the system with it. "Absorption, so deadly in all other circumstances, is thus converted into the means of safety. It searches for the poison in the depths of the tissues, and transports it into the veins, to be thence expelled from the body." The general venous plethora is in another respect advantageous: It enables us to transmit, without fear of syncope, a considerable quantity of blood through the poisoned tissue, so as to clear it of the remains of the poison by means, as it were, of an internal washing."—*Am. Med. Recor.*

10. *Case of Death caused by the external application of Opium.*—The application of opium in considerable quantity, to the skin when deprived of its cuticle, is a practice by no means free from danger; and when it is resorted to for allaying irritation, it is necessary to watch the patient carefully. We are acquainted with an instance in which deep coma was induced by the application of an opium poultice to the scrotum previously deprived of the cuticle by a blister, and which would in all likelihood have proved fatal, had not one of the patient's friends called accidentally to see him, and discovered the cause of the state of sopor. The following is an example of death originating in the same source, although the skin was not deprived of its cuticle. A soldier, thirty two years of age, was attacked with phlegmonous erysipelas of the fore and outer part of the right leg, on account of which a linseed poultice, moistened with *fifteen drops* of laudanum, was ordered to be applied to the limb. Next morning he was found in a state of deep sopor, with the face pale, the eyelids tremulous and half open, the pupils contracted, the lips distorted, the muscles of the face affected with spasm, and those of the limbs with convulsions. The surgeon having remarked a strong odour of opium, as well as a yellow coloration of the bandage which enveloped the limb, he had it removed, and found not only the bandage, but all the compresses soaked with laudanum, which was flowing copiously from the poultice. The attendant whose duty it was to make up the poultice, had left it in charge to a hospital servant, without telling him what quantity of laudanum was to be used; and the latter had ignorantly used towards an ounce. Antispasmodics, emetics, and sinapisms, were resorted to; but in vain. The convulsions increased, the pulse became more and more feeble, and the patient died. On inspection, some red points were seen on the arachnoid, a strong opiate odour was exhaled from all parts, and the heart, stomach, and brain, were in a healthy state. In the blood vessels no trace could be found of the poison which had been absorbed.—*Ibid.*

11. *Sounding the Internal Ear in Deafness.*—New improvements and discoveries in practical surgery, are almost daily occurring. Civiale's lithontritia, Dupuytren's vital dilatation of stricture of the urethra, Deguise and Beclard's operation for wounds of the duct of the parotid gland, the restoration of the superpubian, or high operation of lithotomy, Higginbotham's application of nitrate of silver externally in several kinds of inflammation, besides many other processes which it would be too tedious to attempt in this place to enumerate, but which must have been noticed by all the diligent readers of the Recorder, are more than sufficient to demonstrate the flourishing state of this important branch of the healing art.

Where deafness is owing to obstruction of the Eustachian duct and disease of the internal organs of hearing, M. Debau the younger, has succeeded in removing it by inserting a tube, which he calls a sound, into the duct, and through it introducing air in order to distend it, and to expel mucus or other matter. Two cases are related, in which he perfectly succeeded in the operation, and [illegible] restoration of the hearing was complete. One of them was an obstruction of the internal orifice of the Eustachian duct, the sequel of a chronic inflammation, which had frequently reached to the cavity of the tympanum; the second was a chronic inflammation of the Eustachian duct, with contraction. In the first case, the restoration of hearing was instantaneous, as soon as air had been introduced to distend the cavity. In the second patient, several trials were necessary, as it was some time before the end of the instrument, by proper and moderate force, could penetrate into the cavity of the tympanum.

It is obvious, that the most minute anatomical knowledge, and a happy manual dexterity, ought to be possessed by the surgeon who would expect to succeed in introducing the instrument. The operation is asserted to be neither disagreeable nor painful, in any peculiar degree, to the patient.—*Ibid.*

